# Development of a novel TaqMan-based real-time PCR assay for the detection of porcine boca-like virus (Pbo-likeV)

**DOI:** 10.1186/1743-422X-8-357

**Published:** 2011-07-19

**Authors:** Bin Li, Shaobo Xiao, Junjie Ma, Yanling Liu, Li Mao, Libin Wen, Aihua Mao, Xuehan Zhang, Yanxiu Ni, Rongli Guo, Junming Zhou, Zhengyu Yu, Lixin Lv, Xiaomin Wang, Liurong Fang, Huanchun Chen, Kongwang He

**Affiliations:** 1 Institute of Veterinary Medicine, Jiangsu Academy of Agricultural Sciences; Key Laboratory of Animal Diseases, Diagnostics, and Immunology, Ministry of Agriculture; National Center for Engineering Research of Veterinary Bio-products, Nanjing 210014, Jiangsu Province, PR China; 2 Division of Animal Infectious Diseases, State Key Laboratory of Agricultural Microbiology, College of Veterinary Medicine, Huazhong Agricultural University, Wuhan 430070, PR China

## Abstract

The recently discovered porcine boca-like virus (Pbo-likeV) is a member of the *Parvoviridae *family, genus *Bocavirus*, and it is potentially associated with swine disease. Several studies have associated Pbo-likeV with postweaning multisystemic wasting syndrome in pigs, but the full spectrum of clinical disease and the epidemiology of Pbo-likeV infection remain unclear. The availability of rapid and reliable molecular diagnostics would aid future studies of this novel virus. Thus, we developed a sensitive and specific TaqMan-based real-time PCR assay to target the Pbo-likeV NP1 gene. The assay reproducibly detected 20 copies of a recombinant DNA plasmid containing the NP1 gene, with a dynamic range of six orders of magnitude (10^2^-10^7 ^copies). The assay did not cross-react with other animal viruses. Clinical evaluation found that Pbo-likeV was present in Chinese swine herds at a frequency of 44.2% (114/258). Higher infection rates were found in diseased pigs (56.1%, 101/180) compared with healthy pigs (16.7%, 13/78) (*P *< 0.05). Our assay for the diagnosis and quantification of Pbo-likeV was highly sensitive and specific, and should provide a reliable real-time tool for epidemiological and pathogenetic study of Pbo-likeV infection.

## Introduction

Parvoviruses are members of the family *Parvoviridae *and can cause a broad spectrum of diseases in animals [1]. Based on their host range, the family *Parvoviridae *is classified into two subfamilies: the *Parvovirinae *subfamily infects vertebrates, while the *Densovirinae *subfamily infects arthropods. According to the International Committee on Taxonomy of Viruses (ICTV), and recent studies, the subfamily *Parvovirinae *can be divided into six genera: *Parvovirus*, *Erythrovirus, Dependovirus*, *Amdovirus*, *Bocavirus*, and a newly proposed genus, *Hokovirus *[2-5].

Bocaviruses are unique among parvoviruses in the subfamily *Parvovirinae*, which includes the bovine parvoviruses (BPV), canine minute viruses (CnMV), gorilla bocavirus (GBoV), and four species of human bocaviruses (HBoV1-4) [1]. Bocaviruses contain a non-enveloped, autonomously replicating, single-stranded DNA virus genome, of approximately 5 kb [2,6-8]. Like other members of the *Parvoviridae *family, bocaviruses contain the NS1 non-structural protein and the VP1/VP2 structural protein. However, a non-structural NP1 protein encoded by an open reading frame (ORF) in the middle of the genome is a unique structure characterizing bocaviruses, which is absent in most *Parvoviridae *members [2].

In 2009, a novel porcine boca-like virus (Pbo-likeV) was discovered in Swedish pigs with postweaning multisystemic wasting syndrome (PMWS) using random amplification and large-scale sequencing technology [9]. Subsequent studies indicated a high prevalence of this novel Pbo-likeV was in weaning piglets with respiratory tract symptoms. Pbo-likeV has also been detected in healthy pigs in China [10,11].

Pbo-likeV has not been successfully cultured and no serological tests are available, while only conventional PCR assays have been described [11]. In contrast to conventional assays, real-time PCR offers rapid results with potentially increased sensitivity and specificity of detection. It is also less prone to false positive results from amplicon contamination and is more amenable to the quantitative estimation of viral load. Here we report the development of a highly sensitive and specific TaqMan-based real-time PCR assay to target the NP1 gene for the rapid detection and quantitation of Pbo-likeV in clinical specimens.

## Materials and methods

### Design of primers and probes

Conserved regions of the Pbo-likeV NP1 genes were identified by nucleotide sequence alignments (GenBank: GU902967-GU902971, HQ223038, and FJ872544). Primers and TaqMan probes were designed using the Primer Express software (version 2.0; Applied Biosystems, USA) to generate a 77 bp amplicon. The probe was labeled with 5-carboxyfluorescein (FAM) at the 5'-end and N, N, N', N'-tetramethyyl-6-carboxyrhodamine (TAMRA) at the 3'-end. Primer and probe sequences are shown in Table 1.

**Table 1:** Primers and probe used in real-time PCR assay for Pbo-likeV

Gene	Primer-probe	Sequence	Position^a^
NP1	Forward	5'-TCGAGCTATACAACCGAAGAAGAGA-3'	37-61
	Reverse	5'-TGTTTCGGAGATGTCCTTGCT-3'	93-113
	Probe	FAM-5'-CAGCTCTTCGAATCGCCGCTCTCC-3'-TAMRA	63-86

^a^Nucleotide position was designated according to NP1 gene of Pbo-likeV strain JSNJ1 (GenBank accession no. HQ872052)

### Construction of the plasmid DNA standard

The full Pbo-likeV NP1 gene was amplified using the forward (5'-ATGAGTGGGCATCACAGCCACA-3') and the reverse primers (5'-TTATTTTCCAGCTTCAGCTTCTTGC-3') from a PCR-positive specimen. The product was cloned into the plasmid vector, pMD18-T (TaKaRa Biotechnology, Dalian, Japan) and verified by sequencing. The recombinant plasmid pMD18-NP1 was purified using a QIAamp mini-prep kit (Qiagen, Hilden, Germany) and quantified using an ND-1000 spectrophotometer (NanoDrop, Wilmington, DE, USA). Serial 10-fold dilutions of pMD18-NP1 were prepared in 10 mM Tris-EDTA buffer (pH 8.0) and stored at -20°C prior to use in standard curve generation.

### Real-time PCR assay for Pbo-likeV

The real-time PCR assay was performed in a 20 μL reaction mixture containing 2 μL extracted DNA or standard plasmid, 10 μL TaqMan Universal PCR Master Mix (Applied Biosystems), 500 nM each of forward and reverse primer, and 250 nM of probe. Amplification and detection were performed with an ABI Prism 7500HT sequence detection system (Applied Biosystems) under the following conditions: uracil-*N*-glycosylase was activated at 50°C for 2 min, followed by PCR activation at 95°C for 10 min and 45 cycles of amplification (15 s at 95°C and 1 min at 60°C). Analysis of each assay was conducted with Sequence Detector software (version 2.1; Applied Biosystems).

### Sensitivity of the real-time PCR

To determine the detection limit and efficiency of the assay, standard plasmid DNA was used as a template and 10-fold serially diluted in 10 mM Tris-EDTA buffer (pH 8.0) to produce 2.00 × 10^1 ^to 2.00 × 10^7 ^copies μL^-1^. The sensitivity of the real-time PCR was compared with conventional PCR [11].

### Specificity and reproducibility of the real-time PCR

Reactions with the positive-specimen template, different viruses (PRRSV, PCV2, PRV, CSFV, JEV, and PTTV), and negative controls (sterile water), were also performed to determine the specificity of the real-time PCR assay. Each intra- and inter-assay was performed in triplicate on three different days to evaluate reproducibility.

### Clinical specimens

Clinical specimens were collected from different pig farms in China. The samples mainly included sera, lungs, lymph nodes, and tonsils of healthy and diseased pigs, especially weaning piglets with respiratory tract symptoms. Tissue samples were macerated and diluted 1:10 in Dulbecco's Modified Eagle Medium (DMEM), homogenized and centrifuged at 1500 × *g *for 10 min to obtain a cell-free supernatant. All samples were stored at -80°C. Virus DNA was extracted from tissue homogenates (lymph node, tonsil, and lung) or sera using the QIAamp DNA Mini kit (Qiagen, Germany), according to the manufacturer's instructions.

## Results

### Optimization of real-time PCR

Optimization of the real-time PCR reaction components and cycling conditions was conducted using DNA standards and clinical samples with a known Pbo-likeV status. Nucleotide primers and MgCl_2 _were titrated to determine optimum concentrations and different annealing and data acquisition temperatures were also evaluated (data not shown). The optimum conditions were defined as those that gave the maximum fluorescence and the lowest Ct values, in the absence of primer dimers or nonspecific amplification.

### Real-time PCR standard curve and dynamic range

**Figure 1:**
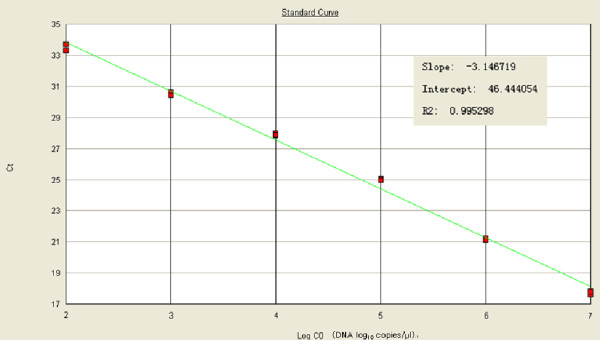
**Linearity of the standard curve in the real-time PCR assay for Pbo-likeV**. The assay was performed using the TaqMan method on serial ten-fold dilutions of the plasmid DNA standard (10^7 ^to 10^2 ^copies). The standard curve produced using pMD18-NP1 was linear, with a correlation co-efficient of 0.995, and a slope of -3.146.

Ten-fold serial dilutions of plasmids were used to construct a standard curve by plotting the logarithm of the plasmid copy number against the measured Ct values (Figure 1). The standard curve had a wide dynamic range of 10^2^-10^7 ^copies μL^-1 ^with a linear correlation (R^2^) of 0.995 between the Ct value and the logarithm of the plasmid copy number.

### Sensitivity of real-time PCR

**Figure 2:**
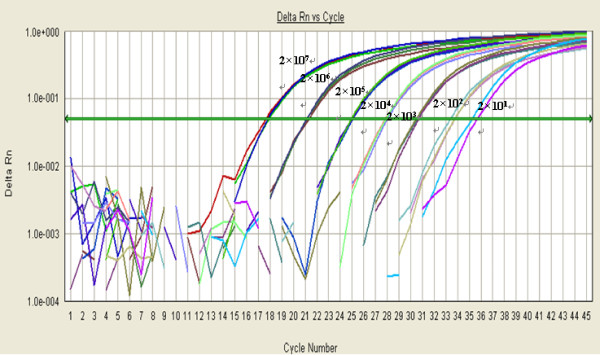
**Sensitivity of the TaqMan real-time PCR assay for Pbo-likeV detection**. Ten-fold dilutions of the standard template pMD18-NP1 containing the target nucleotide sequence were amplified using the real-time PCR assay. Amplification plots of 2.00 × 10^7 ^to 2.00 × 10^1 ^copies/μL of pMD18-NP1 were detected by real-time PCR assay.

The sensitivity of the real-time PCR assay was evaluated by testing ten-fold serial dilutions of the DNA standards (2.00 × 10^1 ^to 2.00 × 10^7 ^copies). Quantitative analysis identified a detection limit of approximately 20 copies of viral DNA (Figure 2).

### Specificity of real-time PCR

**Figure 3:**
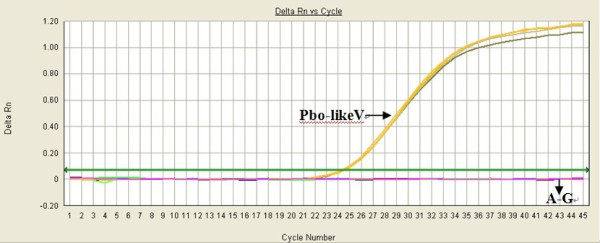
**Specificity of the real-time PCR assay**. Pbo-likeV: Positive sample; A-G: PRRSV, PCV2, CSFV, PRV, JEV, PTTV, and water control.

The specificity of the TaqMan PCR assay was evaluated using eight different reactions, which included, Pbo-likeV, porcine reproductive and respiratory syndrome virus (PRRSV), porcine circovirus type 2 (PCV2), pseudorabies virus (PRV), classic swine fever virus (CSFV), Japanese encephalitis virus (JEV), porcine torque tenovirus (PTTV), and a water negative control. Strong fluorescent signals were obtained from reactions with Pbo-likeV, while the signals from six other porcine virus samples and the water control were equivalent to baseline levels under the optimized reaction conditions (Figure 3). Thus, Pbo-likeV was clearly differentiated from other porcine viruses by comparing the signal strengths at different levels.

### Reproducibility of real-time PCR

The intra- and inter-assay reproducibility was assessed using 10-fold serial dilutions of standard Pbo-likeV plasmid DNA ranging from 2.0 × 10^2 ^to 2.0 × 10^6 ^copies, in triplicate on three different days. Intra-assay variation ranged from 0.35 to 1.62%, while and inter-assay variation ranged from 0.42 to 3.29%, thereby indicating that the real-time PCR was highly reproducible (data not shown).

### Detection of Pbo-likeV in clinical samples by real-time PCR and conventional PCR

Real-time PCR and conventional PCR were performed simultaneously on 258 clinical samples collected from several Chinese swine herds for diagnostic purposes. The results are summarized in Table 2. The total positive frequency of Pbo-likeV in mainland China was 39.9% using conventional PCR. The positive frequency of Pbo-likeV (51.7%, 93/180) in diseased and deceased pigs was significantly higher (12.8%, 10/78) compared with healthy pigs (*P *< 0.05). The results showed that real-time PCR was more sensitive than the conventional PCR assay (Table 2). Furthermore, the positive frequency of Pbo-likeV with real-time PCR (44.2%) was higher than that detected with conventional PCR (39.9%). A high positive frequency of Pbo-likeV (56.1%, 101/180) was also detected in diseased and deceased pigs using real-time PCR. The highest frequencies were observed in lung (69.4%) and lymph samples (54.5%), which also had a high Pbo-likeV viral load (over 10^5 ^copies mg^-1^). We also detected Pbo-likeV in tonsils, which had never been previously reported.

**Table 2:** Detection of Pbo-likeV in pig samples using conventional PCR and TaqMan real-time PCR.

Animal	Health status	Type of tissue or samples	No. positive/no. tested samples (%)	
			
			Conventional PCR	Real-time PCR
	Healthy	Serum	6/50 (12.0)	8/50 (16.0)
		Tonsils	4/28 (14.3)	5/28 (17.9)
		Total	10/78 (12.8)	13/78 (16.7)
				
Pigs	Diseased and deceased	Serum	57/110 (51.8)	61/110 (55.5)
		Tonsils	8/23 (34.8)	9/23 (39.1)
		Lymph	6/11 (54.5)	6/11 (54.5)
		Lung	22/36 (61.1)	25/36 (69.4)
		Total	93/180 (51.7)	101/180 (56.1)
				
	All	Total	103/258 (39.9)	114/258 (44.2)

## Discussion

Bocaviruses are emerging pathogens that cause various diseases in humans and animals. HBoV was first described in 2005 and it causes respiratory diseases in children [6]. However, the animal bocaviruses, CnMV and BPV, were discovered in the 1960s [12]. Pbo-likeV was discovered in 2009 in Swedish pigs with PMWS and it has a close relationship to bocaviruses [9,13,14]. A high prevalence (38.7%) of Pbo-likeV was subsequently associated with PMWS in China [11]. Furthermore, Pbo-likeV was detected in pigs with and without PMWS, with infection rates of 88% and 46%, respectively [10]. Thus, the new Pbo-likeV, and its possible role in swine disease, is currently under intensive investigation.

Methods for the detection of bocaviruses have relied on qualitative, rather than quantitative PCR, which limits the interpretation of results and slows down sample screening. There is an urgent need for rapid and sensitive detection and quantitation assays for Pbo-likeV, both in the pig industry and the research community. In this study, we developed a real-time PCR assay that targets the NP1 gene in the detection of Pbo-likeV. The NP1 gene is highly conserved in bocaviruses, and it is used a as detection target for HBoV [15]. Our assay provides a sensitive (approximately 20 copies) and specific diagnostic tool to allow quantification of Pbo-likeV in clinical specimens. The assay was performed over a wide dynamic range with low intra- and inter-assay variation, and showed no cross-reactivity with other animal viruses. Furthermore, the sensitivity of real-time PCR was significantly higher than that found in conventional PCR assays.

Our investigation of clinical samples indicated that Pbo-likeV was present in Chinese swine herds at a frequency of 44.2% (114/258), while higher infection rates were found in diseased pigs (56.1%, 101/180) compared with healthy pigs (16.7%, 13/78) (*P *< 0.05). We also showed that the Pbo-likeV was more common in PMWS-affected pigs. However, the prevalence of Pbo-likeV in these pigs was lower than that reported previously [10]. The possible reasons for this are as follows. 1) The number of samples was limited. 2) The clinical samples were collected from pigs of all ages, although previous studies found that Pbo-likeV infection rates were low in adult sows and aborted fetuses, whereas weaning piglets were susceptible to infection with Pbo-likeV. 3) Zhai *et al. *[11] detected a seasonal prevalence of Pbo-likeV in swine herds, whereas we collected clinical samples throughout the year.

Pbo-likeV was also usually detected in diseased animals with co-infecting viruses (9-11) and additional studies are required to investigate the presence of Pbo-likeV in pigs and its possible role in PMWS.

## Conclusions

The TaqMan real-time PCR assay described here was a rapid, sensitive, specific, and reproducible method for the detection and quantification of Pbo-likeV. This assay should provide a useful tool for analyzing the clinical and molecular epidemiology of Pbo-likeV infections in swine populations.

## Competing interests

The authors declare that they have no competing interests.

## Authors' contributions

LB was responsible for the study design, sampling, interpretation of data, and drafting the manuscript; SBX, JJM, YLL, LM, LBW, AHM, XHZ, YXN, RLG, JMZ, YZY, LXL, XMW, LRF, HCC, and KWH contributed to experiments and drafting of the manuscript. All authors read and approved the final manuscript.
